# *Nlrp1b1* negatively modulates obesity-induced inflammation by promoting IL-18 production

**DOI:** 10.1038/s41598-019-49546-7

**Published:** 2019-09-25

**Authors:** Jonathan Salazar-León, Ana Laura Valdez-Hernández, Sara García-Jiménez, Luis Román-Domínguez, Enrique Huanosta-Murillo, Laura C. Bonifaz, Leonor Pérez-Martínez, Gustavo Pedraza-Alva

**Affiliations:** 1 Laboratorio de Neuroinmunobiología, Departamento de Medicina Molecular y Bioprocesos, Instituto de Biotecnología, Universidad Nacional Autónoma de México, Cuernavaca, Mor. 62210 Mexico; 2 Facultad de Farmacia, Universidad Autónoma del Estado de Morelos, Cuernavaca, Mor. 62210 Mexico; 3 Unidad de Investigación Médica en Inmunoquímica, Centro Médico Nacional Siglo XXI, Instituto Mexicano del Seguro Social, Ciudad de México, 06720 Mexico

**Keywords:** Inflammasome, Type 2 diabetes

## Abstract

Obesity-induced inflammation, triggered by lipid-mediated activation of the Nlrp3 inflammasome, results in glucose metabolism alterations and type 2 diabetes. This knowledge has been generated using animals deficient for any of the different components of this inflammasome (Caspase-1, Asc or Nlrp3) in the C57BL/6 background. Unlike C57BL/6 mice, which carry allele 2 of the *Nlrp1b* gene (*Nlrp1b2*), Balb/c mice that carry allele 1 (*Nlrp1b1*) are less prone to develop alterations in the glucose metabolism when fed with a high fat diet. However, the molecular bases for these metabolic differences are unknown. Here we show that the *Nlrp1b1* allele down regulates the adipose tissue inflammatory response attenuating glucose intolerance and insulin resistance in obese C57BL/mice. Our results indicate that the positive effects of the Nlrp1b1 inflammasome on glucose tolerance and insulin sensitivity involve IL-18-mediated effects on lipolysis, pointing out that differential expression of allelic variants of genes coding for inflammasome components might control susceptibility or resistance to develop diabetes in obese individuals.

## Introduction

Obesity is a global public health problem resulting from a chronic imbalance between consumption and energy expenditure. The metabolic alterations observed in different organs (pancreas, adipose tissue, liver, heart and skeletal muscle) of obese individuals promote the development of type 2 diabetes (T2D). Recently, it has been shown that chronic inflammation is critical in the pathogenesis of obesity (Reviewed in^1^). Inflammation is promoted by the pathological growth and death of adipocytes in the adipose tissue that results in macrophages infiltration of the adipose tissue^2^. It is though that macrophages phagocyte death adipocytes and that the excess of ingested lipids promotes the production and secretion of inflammatory cytokines such as Interleukin-1β (IL-1β)^3^ that contribute to insulin resistance (IR) and the development of T2D^4,5^. Secretion of these cytokines in response to lipid excess depends on the activation of caspase-1, which is achieved by the formation of a multiprotein complex known as inflammasome. It has been postulated that the NACHT, LRR and PYD domains-containing protein 3 (Nlrp3) inflammasome is responsible for the inflammation that leads to the development of T2D in obese animals, as Nlrp3 deficient obese animals showed a lower degree of IR than wild type (Wt) obese animals^3,6^. However, this knowledge has been generated using animals deficient for the different components of the inflammasome (Caspase-1, Asc or Nlrp3) in the C57BL/6 background. Interestingly, different mouse strains show differences in susceptibility to develop metabolic syndrome when feed with a high fat diet. This correlates with the expression of different alleles of *Nlrp1b* gene, for instance, mice of the C57BL/6 strain expressing allele 2 are susceptible to developing impaired glucose tolerance and IR in response to a hypercaloric diet; however, mice of the Balb/c strain, expressing allele 1 are resistant to develop alterations in glucose metabolism^7,8^. According with this, it has been documented that the Nlrp1b1 and Nlrp1b2 inflammasomes respond differently to the same stimulus. The anthrax lethal toxin activates the Nlrp1b1 but not the Nlrp1b2 inflammasome, resulting in Balb/c macrophages death by pyroptosis while having no effect on C57BL/6 macrophages^9^. Taking this into consideration and that mice deficient for the three alleles of the *Nlrp1* gene (*Nlp1a*, *Nlrp1b* and *Nlrp1c*) develop obesity and metabolic syndrome^10^ we hypothesized that Nlrp1b1 inflammasome may negatively modulate lipid-induced Nlrp3-mediated inflammation and obesity-related metabolic alterations.

Here we show that the *Nlrp1b1* allele controls adipose tissue inflammation as well as improved glucose metabolism in response to energy excess resulting from a high fat diet. Our results indicate that the Nlrp1b1 inflammasome promotes interleukin 18 (IL-18) maturation in the adipose tissue, resulting in lipolysis and reduced dyslipidemia in obese mice.

## Results

### The *Nlrp1b1* allele attenuates obesity-induced alterations on glucose metabolism

**Figure 1:**
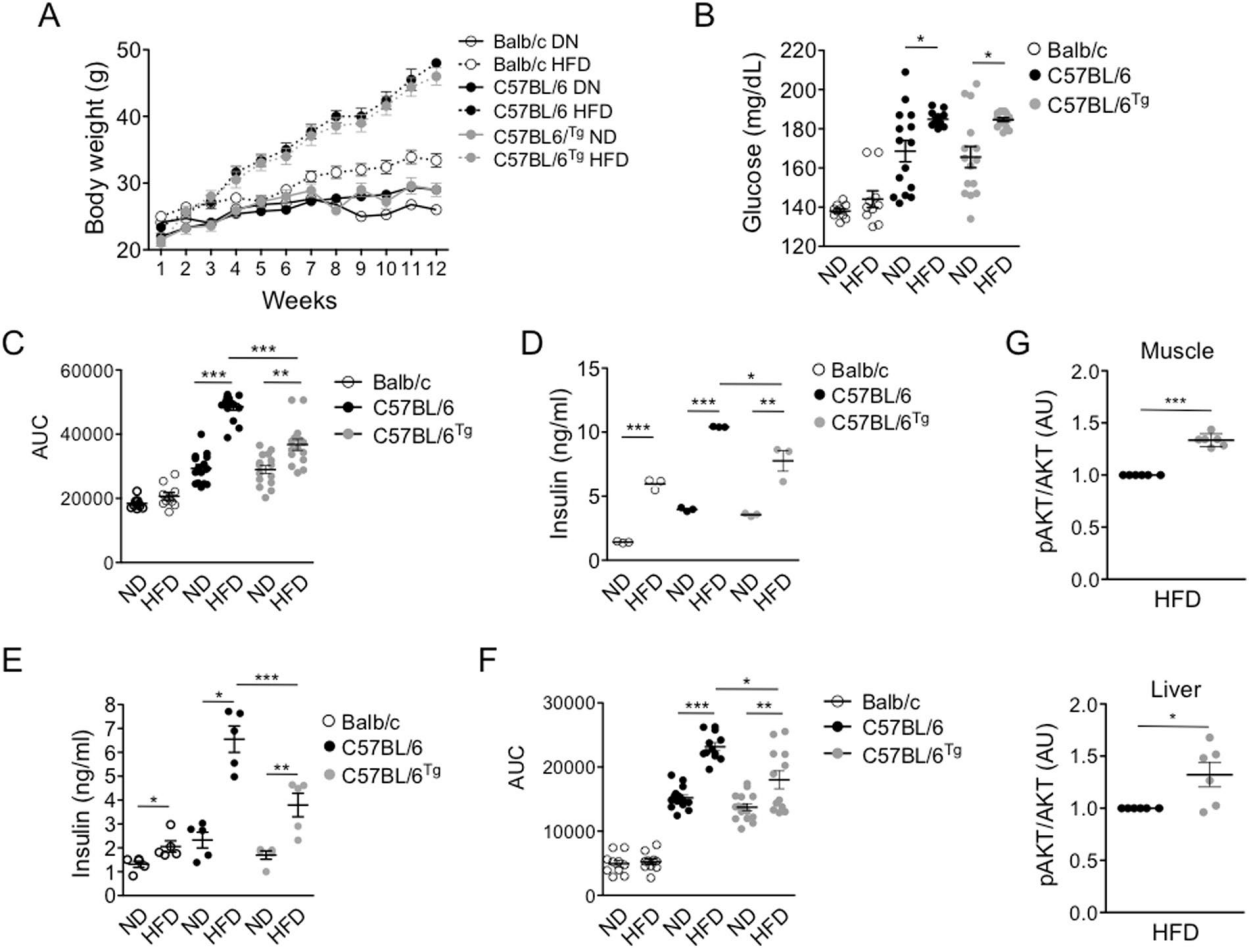
The *Nlrp1b1* gene attenuates obesity-induced glucose tolerance and improves insulin sensitivity in obese mice. (**A**) Mice were fed with regular chow (ND) or with high fat diet (HFD) for three months; body weight was measured every week. (**B**) Mice from the different strains were starved for six hours and basal blood glucose levels were determine as described under material and methods. (**C**) For GTT, starved mice received 1.8 mg of D-glucose per gram of body mass via intraperitoneal and glucose levels were determined at the different time points after glucose administration. The area under the curve (AUC) is shown. (**D**) Insulin secretion after glucose administration was evaluated by ELISA in the sera from starved mice that received 1.8 mg of D-glucose per gram of body mass via intraperitoneal. (**E**) Insulin basal levels were measured in the plasma obtained from starved mice. (**F**) For ITT, after six hours starvation, mice received insulin (1 mU/gr of body mass) via intraperitoneal and glucose levels were determined at the different time points after insulin administration. The area under the curve (AUC) is shown. (**G**) Insulin sensitivity was evaluated indirectly determining the levels of phosphorylated AKT (pAKT) by immunoblot using total cell extracts from liver (upper panel) and muscle (lower panel) and specific antibodies. The normalized (pAKT/Total AKT) densitometry values are shown. The standard error of the mean is shown in the graphs. Balb/c n = 10, Wt (C57BL/6) n = 15 and *Nlrp1b1*-Tg (C57BL/6^tg^) n = 15 p < 0.05 (*); p < 0.01 (**) and p < 0.0001 (***).

Given that inflammasome activation in response to high fat diet contributes to altered glucose metabolism and T2D^3,6^; that different alleles of the *Nlrp1b* gene encode for proteins that respond differently to the same stimulus^9^; that unlike Balb/c mice which carry the *Nlrp1b1* allele, the C57BL/6 mice (Wt) which carry the *Nlrp1b2* allele, are susceptible to develop altered glucose metabolism when fed with a high fat diet^8^; we evaluated whether the expression of the *Nlrp1b1* allele attenuates the negative effect of a high fat diet on the glucose metabolism. As previously reported^9^, the expression of the *Nlrp1b1* allele in a C57BL/6 background (*Nlrp1b1*-Tg), renders macrophages susceptibility to cell death by the anthrax lethal toxin compared to transgenic negative littermate controls animals (Wt C57BL/6), as determined by propidium iodine incorporation (Supplementary Fig. 1A,B) and the release of LDH to the culture supernatant (Supplementary Fig. 1C). Thus, confirming that the Nlrp1b1 inflammasome is functional in C57BL/6 background and activates caspase-1 in response to the anthrax lethal toxin. The expression of the *Nlrp1b1* allele in a C57BL/6 background did not change fasting plasma glucose levels in animals fed with a normal diet (ND) respect to Wt mice (Supplementary Fig. 2A). Likewise, similar glucose tolerance (Supplementary Fig. 2B) and insulin sensitivity (Supplementary Fig. 2C) between *Nlrp1b1*-Tg and Wt C57BL/6 mice were observed. Thus, the *Nlrp1b1* allele in the C57BL/6 background did not alter glucose metabolism in young mice. As previously demonstrated, the Balb/c mice showed lower fasting plasma glucose levels (Supplementary Fig. 2A) and a better control of glucose levels in the circulation compared to Wt C57BL/6^7,8^, or *Nlrp1b1*-Tg mice (Supplementary Fig. 2B). Then, we fed Balb/c, Wt C57BL/6 or *Nlrp1b1*-Tg mice with a ND or with a high fat diet (HFD) for 3 months. Although, there was no difference in weight gain between animals feed with the ND (Fig. 1A), Balb/c mice showed a modest weight gain when fed with the HFD compared with the weight of the Wt C57BL/6 or the *Nlrp1b1*-Tg mice fed with the HFD (Fig. 1A); despite the fact that Balb/c mice ingested more calories than Wt C57BL/6 or *Nlrp1b1*-Tg mice regardless of diet (Supplementary Fig. 3). As well, there was no difference in the food intake between Wt C57BL/6 or *Nlrp1b1*-Tg mice regardless of the diet (Supplementary Fig. 3) indicating that the *Nlrp1b1* allele does not affect food intake and weight gain.

According to previously published data^7,8^, Balb/c mice did not show alterations in the fasting plasma glucose levels when fed with the HFD (Fig. 1B). In contrast, the fasting plasma glucose levels of the Wt C57BL/6 or *Nlrp1b1*-Tg mice were elevated when fed with the HFD compared to those observed in mice feed with the ND (Fig. 1B). Again, there were no differences in fasting plasma glucose levels between Wt C57BL/6 and *Nlrp1b1*-Tg mice fed with the ND or with the HFD (Fig. 1B). As determined by the glucose tolerance test (GTT), Balb/c mice fed with the HFD were glucose tolerant (Fig. 1C and Supplementary Fig. 4A), while the Wt C57BL/6 mice became glucose intolerant when fed with the HFD (Fig. 1C). Interestingly, the *Nlrp1b1*-Tg mice fed with the HFD were less glucose intolerant that Wt C57BL/6 mice fed with the HFD (Fig. 1C), despite the fact that when fed with the ND there were no differences in their glucose tolerance (Fig. 1C). The difference in glucose tolerance between Wt C57BL/6 and *Nlrp1b1*-Tg mice did not result from defects in insulin secretion in response to glucose administration, since Wt C57BL/6 mice fed with the HFD secreted more insulin than *Nlrp1b1*-Tg mice fed with the HFD (Fig. 1D). Likewise, WT C57BL/6 and *Nlrp1b1*-Tg animals fed with the ND secrete similar insulin levels in response to glucose (Fig. 1D), suggesting that the *Nlrp1b1* allele improves insulin sensitivity thus preventing IR in response to a HFD. In agreement with this, fasting insulin levels in the Wt C57BL/6 mice fed with the HFD were increased in comparison with the levels of mice fed with the ND or with the levels observed in the Balb/c mice fed with the HFD (Fig. 1E). Consistently, the *Nlrp1b1*-Tg mice fed with the HFD showed reduced basal insulin levels when compared with those of the Wt C57BL/6 mice (Fig. 1E) but higher levels than those of the Balb/c mice fed with the HFD. Interestingly, the *Nlrp1b1*-Tg mice fed with the ND also showed significantly lower insulin levels that those of the Wt C57BL/6 mice fed with the ND (Fig. 1E). IR was confirmed by the insulin tolerance test (ITT), the HFD induced IR in Wt C57BL/6 but not in Balb/c mice, again, the expression of the *Nlrp1b1* gene in the C57BL/6 background reduced IR respect to the Wt C57BL/6 mice fed with HFD (Fig. 1F and Supplementary Fig. 4B). This was consistent with a more efficient activation of the AKT pathway in response to insulin in the muscle or liver of the *Nlrp1b1*-Tg whitch was significantly more active than in Wt C57BL/6 mice fed with the HFD (Fig. 1G and Supplementary Fig. 4C). Importantly, the glucose tolerance and insulin sensitivity improvement showed in the *Nlrp1b1*-Tg mice fed with the HFD did not result from nonspecific effects due to the integration site of the DNA fragment used to generate the transgenic mice, since similar results were obtained using two different transgenic lines (Supplementary Fig. 5). Together, these results suggest that the *Nlrp1b1* allele attenuates the alterations in glucose metabolism associated to obesity by improving insulin sensitivity.

### The *Nlrp1b1* allele attenuates the inflammatory process in the adipose tissue of obese mice

**Figure 2:**
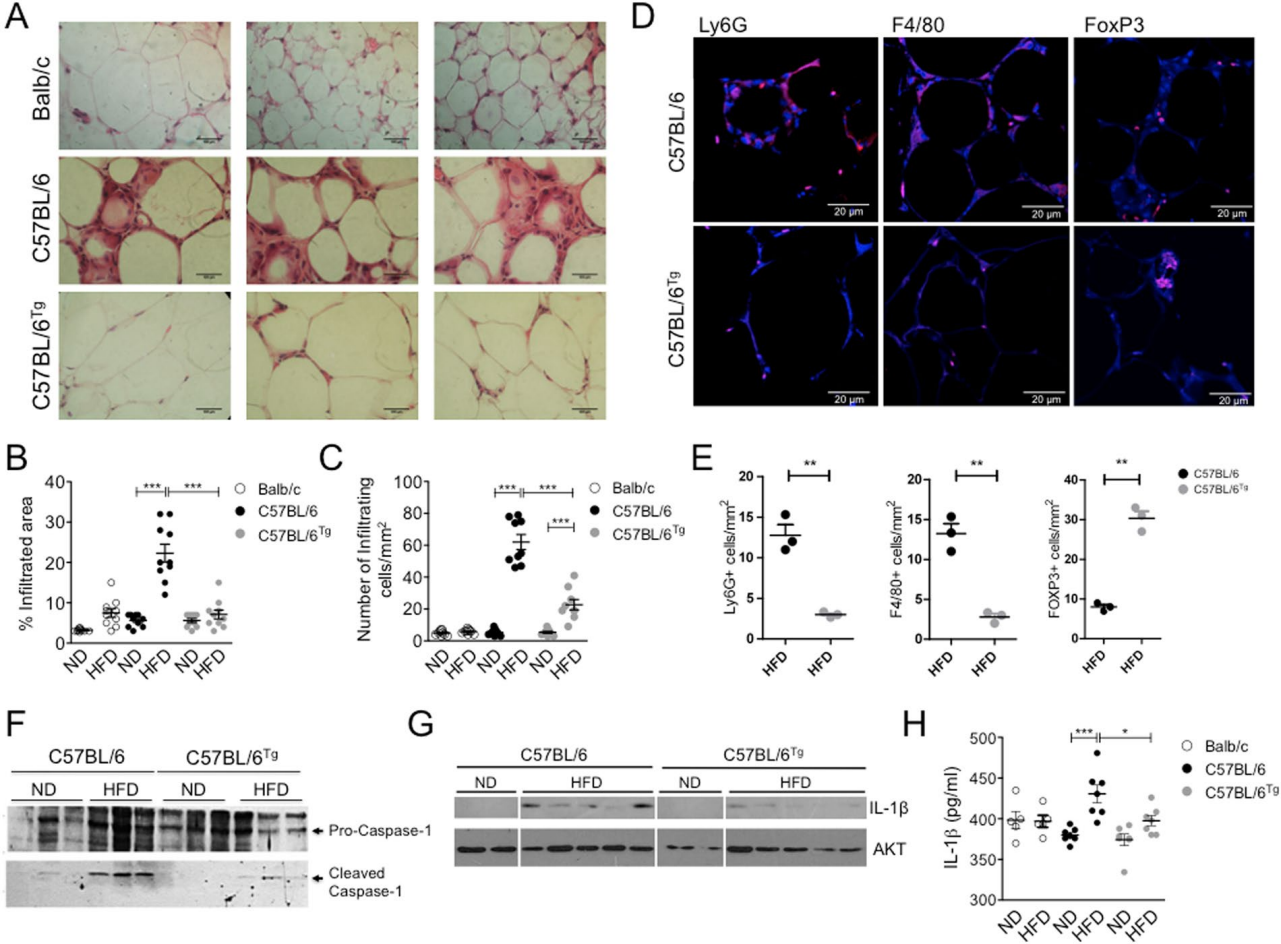
The *Nlrp1b1* gene attenuates the inflammatory process in the adipose tissue resulting from a HFD consumption. Mice were fed with regular chow (ND) or with a high fat diet (HFD) for three months. Mice were sacrificed and peritoneal adipose tissue was collected, snap frozen or fixed in paraformaldehyde. (**A**) The cellular infiltrated was evaluated in adipose tissue sections, from mice fed with the HFD, stained with hematoxylin and eosin under light microscopy. Bar represents 100 μM. (**B**) The infiltrated areas as well as the number of infiltrating cells (**C**) were determined using ImageJ software as described under materials and methods. The standard error of the mean is shown in the graphs. Balb/c n = 5, Wt (C57BL/6) n = 5 and *Nlrp1b1*-Tg (C57BL/6^tg^) n = 5 of each diet condition. p < 0.0001 (***). (**D**) Peritoneal adipose tissue was collected and included in paraffin. The infiltrating cells were evaluated in adipose tissue sections stained with antibodies against Ly6G (AF 488), F4/80 (AF 594) and FoxP3 (AF 647) and visualized under confocal microscope. (**E**) The infiltrated area as well as the number of infiltrating cells were determined using ImageJ software. Scale bar represents 20 μm. Graphs represent mean ± standard deviation. Wt (C57BL/6) n = 3 and *Nlrp1b1*-Tg (C57BL/6^tg^) n = 3 fed with the HFD. p < 0.01 (**). (**F**) Cleaved caspase-1 and mature IL-1β levels (**G**) were evaluated by immunoblot using adipose total cell extracts and specific antibodies. The levels of the full-length caspase-1 and AKT were determined as loading controls, respectively. (**H**) Circulating IL-1β levels were quantified by ELISA in the sera from the different mouse strains fed with ND or HFD as indicated under material and methods. Balb/c n = 10, Wt (C57BL/6) n = 15 and *Nlrp1b1*-Tg (C57BL/6^tg^) n = 15 for immunoblot analysis. Balb/c n = 5, Wt (C57BL/6) n = 7 and *Nlrp1b1*-Tg (C57BL/6^tg^) n = 7 for the IL-1β ELISA analysis. p < 0.05 (*), p < 0.01 (**) and p < 0.0001 (***).

To evaluate whether the improved glucose metabolism observed in obese *Nlrp1b1*-Tg mice was due to reduced inflammation in the adipose tissue, we performed a histological analysis (Fig. 2A). Although the adipose tissue of the Balb/c mice fed with the HFD showed an increase in the infiltrated area compared with the mice fed with the ND (Fig. 2B), this was clearly minor than that observed in the adipose tissue of the obese Wt C57BL/6 mice (Fig. 2B,C), and similar to the infiltrated in the adipose tissue of lean Wt C57BL/6 mice (Fig. 2B,C). Albeit the infiltration observed in the adipose tissue of *Nlrp1b1*-Tg mice fed with the HFD was increased in comparison with the infiltrated of lean *Nlrp1b1*-Tg mice (Fig. 2B,C), it was clearly reduced when compared with obese Wt C57BL/6 mice (Fig. 2B,C) and comparable to that of Balb/ mice fed with the HFD (Fig. 2B,C). Accordingly, the adipose tissue of *Nlrp1b1*-Tg mice fed with the HFD showed a significant reduction in the number of infiltrating neutrophils and macrophages when compared with obese Wt C57BL/6 mice (Fig. 2D,E). In stark contrast, the *Nlrp1b1*-Tg mice fed with the HFD contained higher numbers of FoxP3^+^ cells (Fig. 2D,E). These data correlated with the fact that adipose tissue from the *Nlrp1b1*-Tg obese mice showed reduced levels of chemokines involved in neutrophil and macrophage migration like CCL-11, GM-CSF, CXCL9^11,12^ but higher levels of CCL-1 and CCL-5 implicated in TRegs chemotaxis^13–16^, when compared with the levels found in the adipose tissue of obese Wt C57BL/6 mice (Supplementary Fig. 6).

**Figure 3:**
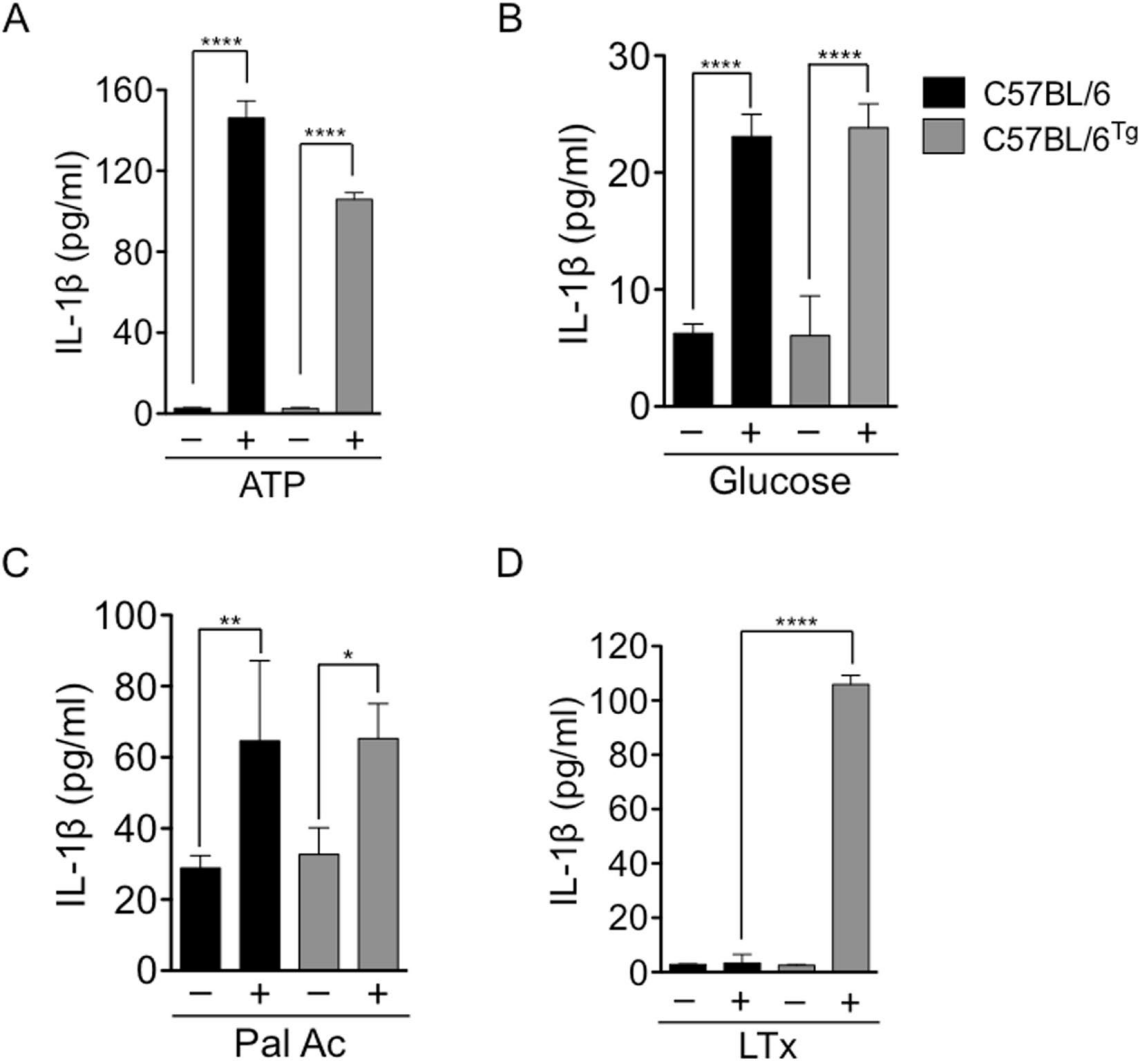
The *Nlrp1b1* gene does not impair Nlrp3 inflammasome activation by metabolic stress-derived danger signals. Bone marrow derived macrophages from the indicated mouse strains were exposed to LPS (100 ng/ml) for 4 hours and then treated with 5 mM ATP for 2 hours (**A**); 30 mM Sorbitol (−) or 30 mM Glucose (+) for 24 hours (**B**); 2% BSA (−) or 2%BSA-400mM Palmitic Acid (Pal Al, +) for 24 hr (**C**), or left untreated (−) or directly exposed to the anthrax lethal toxin (LTx, 200 ng/ml lethal factor and 1 mg/ml protective antigen) for 2 hr (**D**). Culture supernatants were collected and the levels of secreted IL-1β were determined by ELISA. Data represent the mean ± standard deviation of three independent experiments, p < 0.05 (*), p < 0.01 (**) and p < 0.00001 (****).

Congruent with these data, the levels of active caspase-1 in the adipose tissue were increased in Wt C57BL/6 mice fed with the HFD when compared with the levels observed in lean C57BL/6 mice adipose tissue (Fig. 2F, lower panel). Active caspase-1 levels were also increased in obese *Nlrp1b1*-Tg mice but to a lesser extent that those observed in obese Wt C57BL/6 mice (Fig. 2F, lower panel). In agreement with this, the levels of mature IL-1β levels were also reduced in the adipose tissue (Fig. 2G, upper panel and Supplementary Fig. 6) and in the plasma (Fig. 2H) of the *Nlrp1b1*-Tg mice fed with the HFD, when compared with those of obese Wt C57BL/6 mice fed with the HFD. Interestingly, the Balb/c mice did not show an increase in IL-1β levels in circulation when fed with a HFD (Fig. 2H). The differences in IL-1β levels observed between Wt C57BL/6 and obese *Nlrp1b1*-Tg mice did not result from negative effects on Nlrp3 inflammasome activation due to *Nlrp1b1* overexpression, since bone marrow derived macrophages from Wt C57BL/6 or *Nlrp1b1*-Tg mice underwent pyroptosis similarly in response to LPS and ATP treatment (Supplementary Fig. 7A). Furthermore, activation of the Nlrp3 inflammasome in Wt C57BL/6 or *Nlrp1b1*-Tg bone marrow derived macrophages in response to ATP (Fig. 3A) or danger signals associated with obesity like glucose (Fig. 3B) or palmitic acid (Fig. 3C) resulted in similar levels of IL-1β secreted to the culture supernatant. While the Nlrp1b1 inflammasome was activated only in *Nlrp1b1*-Tg bone marrow derived macrophages upon exposure to the anthrax lethal toxin (Fig. 3D). Additionally, in agreement with the fact that the *Nlrp1b1-transgenic* mice are maintained as heterozygotes, the *Nlrp1b1* copy number was not above the diploid *Nlrp1b* gene copy number found in Balb/c mice (Supplementary Fig. 7B,C).

According with the reduced levels of mature IL-1β in the adipose tissue and in circulation of the *Nlrp1b1*-Tg mice, the levels of inflammatory cytokines like TNF and IL-6 were also reduced in the adipose tissue of the *Nlrp1b1*-Tg mice compared with those observed in the adipose tissue of obese Wt C57BL/6 mice (Supplementary Fig. 6). Furthermore, Leptin levels were also reduced in the adipose tissue of the *Nlrp1b1*-Tg mice fed with the HFD compared with those observed in the adipose tissue of the obese Wt C57BL/6 (Supplementary Fig. 6), which is in agreement with the role of Leptin as an inflammatory cytokine (Reviewed in^17^). In contrast, the levels of anti-inflammatory cytokines like IL-10 and IL-4 were increased in the adipose tissue of obese *Nlrp1b1*-Tg mice compared with the levels of obese Wt C57BL6 mice (Supplementary Fig. 6). Together, these results indicate that the *Nlrp1b1* allele reduced the inflammatory process resulting from the HFD in the adipose tissue.

### The *Nlrp1b1* allele improves lipid metabolism in obese mice

**Figure 4:**
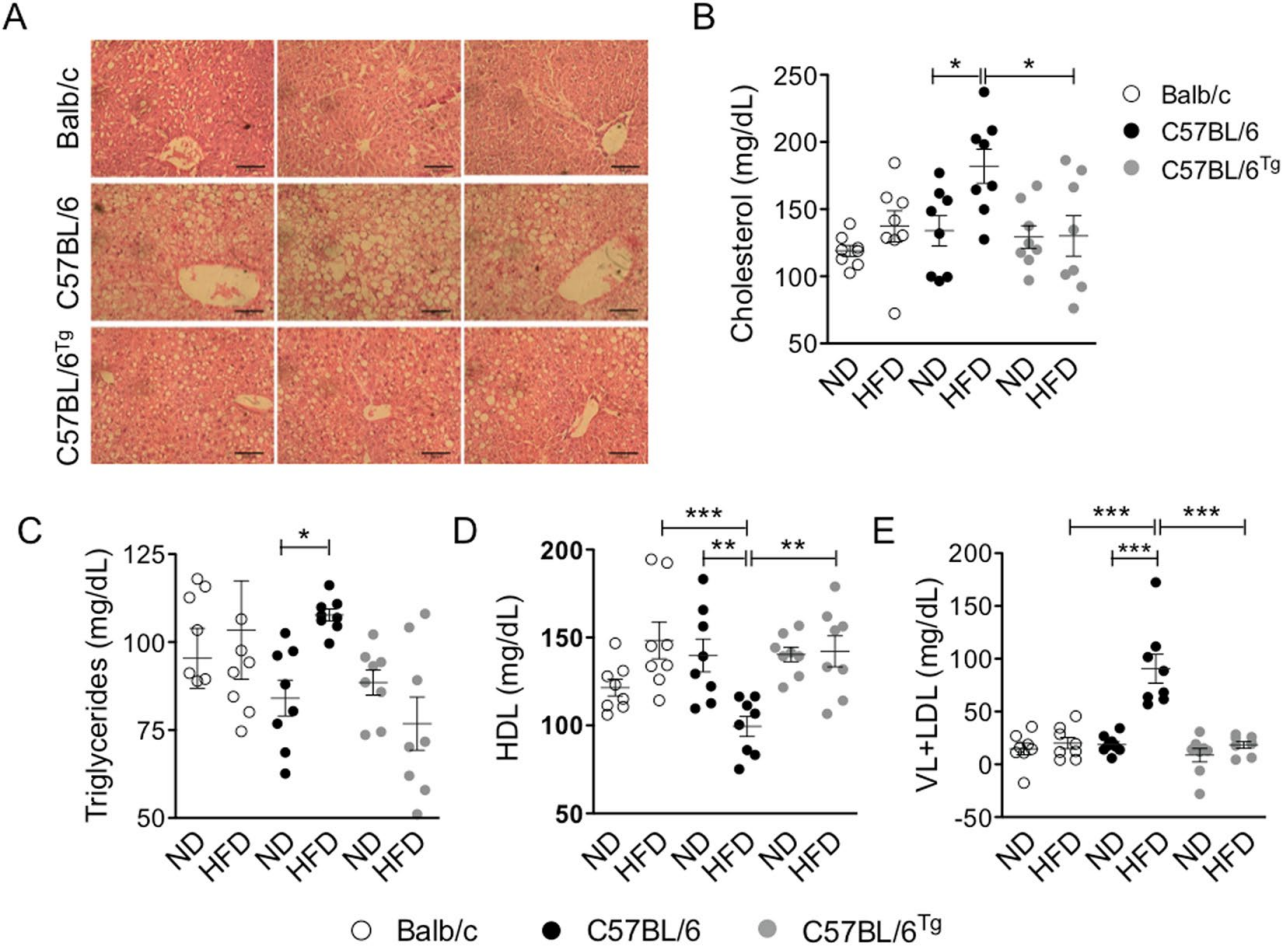
The *Nlrp1b1* gene improves lipid metabolism in obese mice. Mice were fed with regular chow (ND) or with a high fat diet (HFD) for three months. Mice were sacrificed and the liver was fixed in paraformaldehyde. (**A**) Hepatic steatosis was evaluated in tissue sections, from mice fed with the HFD, stained with hematoxylin and eosin under light microscopy. (**B**) Cholesterol, (**C**) Triglycerides and (**D**) HDL levels were quantified in the plasma as described under materials and methods. (**F**) Levels of LDL were calculated using Anandaraja’s formula as described under materials and methods. Scale bar represents 100 μm. Data were analyzed by one-way ANOVA followed by Tukey’s multiple comparison test. The standard error of the mean is shown in the graphs. Balb/c n = 10, Wt (C57BL/6) n = 10 and *Nlrp1b1*-Tg (C57BL/6^tg^) n = 10. p < 0.05 (*), p < 0.01 (**) and p < 0.0001 (***).

In agreement with the fact that the inflammatory process in the adipose tissue resulting from a HFD promotes hepatic steatosis^1^, we observed increased hepatic steatosis in obese Wt C57BL/6 mice compared to that observed in obese *Nlrp1b1*-Tg mice or Balb/c mice fed with the HFD (Fig. 4A). These results suggest that the *Nlrp1b1* allele could improve lipid metabolism in obese mice, therefore we evaluated circulating free fatty acid levels. Although the cholesterol levels were similar in all mice groups fed with the ND or the HFD, Wt mice C57BL/6 showed slightly increased cholesterol levels when fed with the HFD, this increment was not observed neither in the *Nlrp1b1*-Tg mice nor in Balb/c mice fed with the HFD (Fig. 4B). Likewise, triglycerides levels were increased only in Wt C57BL/6 mice fed with the HFD (Fig. 4C). In contrast, the HDL levels were reduced in Wt C57BL/6 mice fed with the HFD (Fig. 4D), while the HDL levels in the *Nlrp1b1*-Tg or Balb/c mice did not change with the diet (Fig. 4D). Accordingly, we observed a significant increase in LDL levels in obese Wt C57BL/6 mice only (Fig. 4E). Together these data indicate that the *Nlrp1b1* allele reduces dyslipidemia in obese mice.

### The *Nlrp1b1* allele enhances IL-18 production in the adipose tissue of obese mice

**Figure 5:**
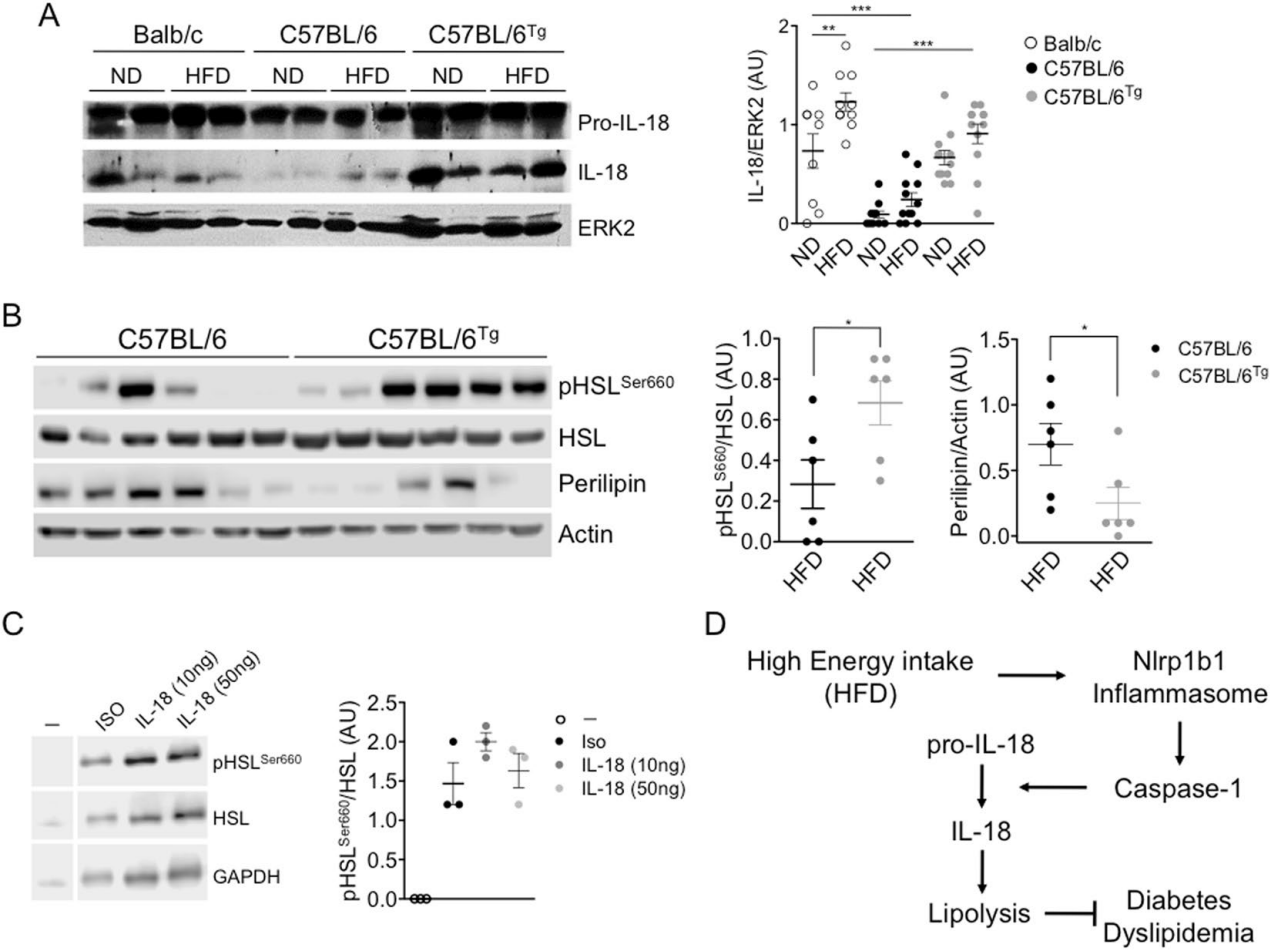
The *Nlrp1b1* gene promotes IL-18 maturation and lipolysis in the adipose tissue of obese mice. Mice were fed with regular chow (ND) or with a high fat diet (HFD) for three months. Mice were sacrificed and peritoneal adipose tissue was collected and snap frozen. (**A**) Levels of mature IL-18 were evaluated by immunoblot using adipose total cell extracts and specific antibodies. ERK2 levels were used as loading control. Normalized IL-18/ERK densitometry values are shown. Data were analyzed by one-way ANOVA followed by Tukey’s multiple comparison test. The standard error of the mean is shown in the graphs. Balb/c n = 8, Wt (C57BL/6) n = 10 and *Nlrp1b1*-Tg (C57BL/6^tg^) n = 10. p < 0.01 (**) and p < 0.0001 (***). (**B**) Levels of phosphorylated HSL at Ser^660^ (pHSL), HSL levels and Perelipin levels were evaluated by immunoblot using adipose total cell extracts and specific antibodies. Actin levels were used as loading control. Normalized pHSL/HSL/Actin and Perilipin/Actin densitometry values are shown. Data were analyzed by one-way ANOVA followed by Tukey’s multiple comparison tests. The standard error of the mean is shown in the graphs. Wt (C57BL/6) n = 6 and *Nlrp1b1*-Tg (C57BL/6^tg^) n = 6. p < 0.05 (*). (**C**) 3T3-L1 derived adipocytes were left untreated (−) or treated with 10 μM isoproterenol (ISO), or with recombinant IL-18 (IL-18; 10 ng/ml or 50 ng/ml) for 15 minutes. Cells were harvested and protein extracts prepared. The levels of phosphorylated HSL at Ser^660^ (pHSL) and total HSL were evaluated by immunoblot using specific antibodies. GAPDH levels were used as loading control. A representative blot of three independent experiments (left panel) and the normalized pHSL/HSL densitometry values are shown (right panel). (**D**) The Nlrp1b1 receptor by a yet unknown mechanism is activated in response to energy excess resulting in the formation of the Nlrp1b1 inflammasome that preferentially results in IL-18 maturation. In turn, IL-18 promotes lipolysis attenuating the metabolic alterations in obese mice.

Recently it was shown that the Nlrp1 inflammasome, through the maturation of IL-18 in the adipose tissue, regulates the development of obesity and metabolic syndrome resulting from a rich caloric diet in C57BL/6 mice, by promoting lipolysis^10^. Therefore, we speculated that the *Nlrp1b1-*transgenic mice produce more IL-18 in the adipose tissue. Indeed, Wt C57BL/6 mice showed reduced levels of mature IL-18 when compared with the levels observed in the *Nlrp1b1*-Tg animals or Balb/c mice, independently of the diet (Fig. 5A); however, mature IL-18 levels were further increased in both the *Nlrp1b1*-Tg and Balb/c mice fed with the HFD (Fig. 5A). In agreement with the fact that phosphorylation of Hormone-sensitive lipase (HSL) at Ser^660^ activates lipolysis^18^, we found that the levels of phosphorylated HSL on Ser^660^ were higher in the adipose tissue of the *Nlrp1b1*-Tg animals than those observed in the adipose tissue of the Wt C57BL/6 mice (Fig. 5B). Moreover, congruent with data indicating that the levels of perilipin, a protein associated to the surface of lipid droplets, decreased when lipolysis is activated^19,20^ we found reduced perilipin levels in the adipose tissue of obese *Nlrp1b1*-Tg animals when compared with the levels found in the Wt C57BL/6 mice fed with the HFD (Fig. 5B). To confirm that IL-18 promotes lipolysis, we exposed 3T3-L1 adipocytes to IL-18 and evaluated the HSL phosphorylation levels on Ser^660^. As expected, in response to isoproterenol, a β-Adrenergic receptor agonist, HSL phosphorylation on Ser^660^ was induced 15 min after treatment (Fig. 5C). Likewise, IL-18 promoted HSL phosphorylation on Ser^660^ independently of the concentration used (10 ng/ml or 50 ng/ml) (Fig. 5C). Consistent with the data indicating that the effect of the *Nlrp1* genes on lipids and glucose metabolism is independent from their hematopoietic expression^10^, Wt C57BL/6 lethally irradiated and reconstituted with bone marrow from the *Nlrp1b1*-Tg mice fed with the HFD for three months (Supplementary Fig. 8A) did not show differences in food intake (Supplementary Fig. 8B), weight gain (Supplementary Fig. 8C) or glucose tolerance (Supplementary Fig. 8D), when compared with mice reconstituted with Wt C57BL/6 bone morrow and fed with the HFD (Supplementary Fig. 8). Together, these results indicate that in response to a caloric excess, the Nlrp1b1 inflammasome produces more IL-18 than the Nlrp1b2 inflammasome also, that the IL-18 elevated levels sustain lipolysis thus reducing adipose tissue inflammation and improving insulin sensitivity.

## Discussion

The inflammatory response observed in the adipose tissue in response to lipid accumulation is mediated by the Nlrp3 inflammasome, which promotes caspase-1 activation and IL-1β production^3^. The chronic inflammatory process initiated in the adipose tissue leads to IR, impairing the ability of the liver and the muscle to uptake glucose and thus resulting in increased circulating glucose levels. Additionally, inflammatory cytokines also affect insulin and leptin signaling in the hypothalamus impairing the control of food intake and energy expenditure^21^. Together, insulin and leptin resistance eventually lead to the development of T2D.

Unlike Nlrp3, the role for other Nlrp receptors as metabolic sensors is largely unknown. Here, we tested the idea that differential activation of the Nlrp1b1 inflammasome by lipid excess could modulate the inflammatory response in the adipose tissue and the susceptibility to develop alterations in the glucose metabolism in obese mice.

According with our hypothesis, transgenic mice expressing the *Nlrp1b1* allele in a C57BL/6 background, which endogenously carry the *Nlrp1b2* allele, showed reduced glucose intolerance and improved insulin sensitivity when fed with a HFD despite the fact that they gained the same weight than Wt C57BL6/ mice. However, when compared with Balb/c mice, the *Nlrp1b1*-transgenic mice fed with a HFD showed glucose intolerance and reduced insulin sensitivity, thus indicating that there are other factors in the Balb/c background playing an important role to maintaining glucose homeostasis despite the energy excess provided by the HFD. In this regard, alterations in the microbiome after feeding with a HFD is a factor leading to the development of T2D^22^. Recently, it has been shown that Nod2 is required to maintain a normal microbiome in Balb/c mice when fed with a HFD and thus protecting them from obesity and metabolic syndrome since, Balb/c mice lacking Nod2 fed with a HFD gain weight and present glucose intolerance and IR similarly to C57BL/6 mice^23^. This phenotype resulted from alterations in the microbiome, which was similar to that of C57BL/6 mice^23^. Interestingly, Nod2 and Nlrp1b interact in response to muramyl dipeptide to promote caspase-1 activation and IL-1β production^24^. Hence, it is possible that the interaction between Nod2 and the Nlrp1b2 inflammasome (C57BL/6) is not as efficient, as the interaction of Nod2 with the Nlrp1b1 inflammasome (Balb/c), in promoting caspase-1 activation and IL-1β production in the colon, to prevent dysbiosis in response to a HFD, thus facilitating glucose metabolism alterations, dyslipidemia and ultimately T2D. Nonetheless, this idea needs to be tested experimentally.

The reduction in glucose intolerance and improved insulin sensitivity observed in the *Nlrp1b1*-transgenic mice relative to Wt C57BL/6 mice, correlated with a clear reduction in the inflammatory process triggered by the HFD in the adipose tissue (reduced cellular infiltrated and reduced caspase-1 activation) which correlated with lower blood IL-1β levels compared with Wt C57BL/6. The reduced number of infiltrating neutrophils and macrophages in the adipose tissue of the *Nlrp1b1*-transgenic mice correlated with the reduction in chemokines levels involved in neutrophils and macrophage migration like CCL11, GM-CSF, CXCL9^11,12^, this clearly differ from that in the adipose tissue of the Wt C57BL/6 mice. In utter contrast, the presence of FoxP3^+^ Tregs was increased in the adipose tissue of the *Nlrp1b1*-transgenic mice compared with the presence of FoxP3^+^ cells observed in the adipose tissue of obese Wt C57BL/6 mice. Again, this correlated with the contrasting levels of chemokines (CCL1 and CCL5) involved in the recruitment of FoxP3^+^ Tregs cells to different tissues^13–16^ among the two mouse strains. Interestingly, it has been shown that IL-18 promotes both the differentiation and the function of FoxP3^+^ Tregs^25–27^ consistent with this observation, the levels of IL-10 were higher in the adipose tissue of the *Nlrp1b1*-transgenic mice than in the adipose tissue of the Wt C57BL/6 mice. IL-18-mediated Tregs activation could explain also that IL-17 levels were higher in the adipose tissue of the obese *Nlrp1b1*-transgenic mice than the levels observed in the obese Wt C57BL/6 mice, since it has been reported that FoxP3^+^ cells produce IL-17 while keeping suppressive functions^28^. The presence of neutrophils and macrophages in the adipose tissue of the obese Wt C57BL7/6 mice correlated with higher levels of inflammatory cytokines like IL-6, TNF and IL-1β. Interestingly, although both mouse strains gained the same weight when fed with the HFD, leptin levels were considerably higher in the adipose tissue of Wt C57BL/6 mice than the levels found in the *Nlrp1b1*-transgenic mice which suggest that this difference could result from the inflammatory environment rather than the metabolic state of the adipose tissue. This is consistent with the fact that leptin functions as an inflammatory cytokine^17^. Although leptin promoter activity is regulated by transcription factors such as AP-1 and NFE2L1 that can be activated by inflammatory signals and regulate the expression of inflammatory cytokines like TNF^29,30^; it is still an open question whether *leptin* gene expression in the adipose tissue is regulated by specific inflammatory signals.

As mentioned, the Nlrp1b1 inflammasome attenuated the inflammatory response in the adipose tissue triggered by the lipid excess consumption resulting from the HFD. The only difference in the architecture of the adipose tissue between the *Nlrp1b1*-transgenic mice and the Balb/c mice fed with the HFD was that the adipocytes in the *Nlrp1b1*-transgenic mice were bigger than those of the Balb/c mice. Opposite results were recently published showing that the adipocytes from Balb/c mice fed with a HFD were bigger that those of C57BL/6 mice^31^; however, in this study the mice were fed for 6 months. These results indicate differences in the mechanism by which both strains deal with the excess of lipids and that this is independent of the Nlrp1b inflammasome. At early stages of obesity C57BL/6 mice promote hypertrophy, while Balb/c seems to promote hyperplasia.

In agreement with our data, recently it was shown that, in contrast to the Nlrp3 inflammasome, the activation of the Nlrp1 inflammasome is required to maintain lipid and glucose homeostasis in response to energy excess^10^. C57BL/6 mice lacking the Nlrp1a, b and c inflammasomes develop more severe alterations in the glucose and lipid metabolism than Wt animals when fed with a high fat or high protein diet^10^. Inasmuch as IL-18 has been involved in energy expenditure^32^, this was attributed to the reduced IL-18 levels in the adipose tissue of obese C57BL/6 *Nlrp1*^−/−^ mice compared to the IL-18 levels found in Wt mice^10^. Mice deficient for IL-18 or IL-18 receptor are susceptible to obesity and to develop glucose intolerance and IR^32,33^. In contrast, IL-18 overexpression protects mice from developing glucose tolerance and IR when fed with a HFD^34^. According with this, we found that the *Nlrp1b1*-transgenic mice showed higher IL-18 protein levels in the adipose tissue than those found in C57BL/6 mice, independently of the diet. Nonetheless, the IL-18 levels were further upregulated in response to the HFD. Strikingly, the IL-18 levels found in the *Nlrp1b1*-transgenic mice were similar to those observed in the adipose tissue from Balb/c mice. The mechanism by which IL-18 promotes energy expenditure is still not yet well defined but involves lipolysis^10,34^. Nonetheless, exposing 3T3-L1 adipocytes to IL-18 resulted in increased levels of active HSL (as determined by HSL phosphorylation levels at Ser^660^)^18^, confirming that IL-18 promotes adipocyte lipolysis. Accordingly, the increased active HSL levels correlated with reduced perilipin levels in the adipose tissue of obese *Nlrp1b1*-transgenic mice compared with those found in obese Wt C57BL/6 mice. In concordance with the IL-18 levels, both Balb/c and *Nlrp1b1*-transgenic mice showed reduced hepatic steatosis and reduced dyslipidemia when fed with the HFD than obese Wt C57BL/6 mice. Together, our results indicate that the Nlrp1b1 inflammasome has a protective function against developing dyslipidemia and T2D in response to energy excess, through efficient IL-18 production (Fig. 5D). These effects did not result from impaired Nlrp3 inflammasome activation in response to molecules associated with metabolic stress such as high glucose or lipid levels, since *Nlrp1b1*-transgenic macrophages secreted similar IL-1β amounts than Wt C57BL/c macrophages when exposed to those metabolic insults. This is consistent with the fact that the positive effects of the *Nlrp1b1* gene on glucose homeostasis are not mediated directly by hematopoietic cells, since *Nlrp1b1*-transgenic bone marrow transplants into recipient Wt C57BL/6 mice did not improved glucose tolerance in mice fed with the HFD, which is in agreement with the fact that *Nlrp1* deficient hematopoietic cells did not promoted alterations in lipids and glucose metabolism when transplanted into Wt C57BL/6 mice^10^.

Given that in the *Nlrp1* deficient mice the *Nlrp1a* and *Nlrp1c* genes were also eliminated^10^ and that these genes are identical among mouse strains, our data together with that of Murphy *et al*.^10^, suggest that i) the *Nlrp1b* gene is involved in attenuating the inflammatory response in the adipose tissue and promoting lipolysis in response to a caloric excess and ii) that the gene products of different alleles of the *Nlrp1b* gene have different efficacies to promote IL-18 production and therefore, to protect from dyslipidemia and T2D in obese animals. Thus, the *Nlrp1b1*-transgenic mice resemble human healthy obese individuals. One wonders whether single nucleotide variant of the human *NLRP1* gene correlates with IL-18 levels and consequently with the susceptibility of obese individuals to develop dyslipidemias and/or T2D.

## Materials and Methods

### Animals

Balb/c mice were from Charles River. C57BL/6 J transgenic mice expressing the *Nlrp1b1* allele have been previously described^9^ and were kindly donated by Dr. Cory Teuscher from the Immunobiology Division, Department of Medicine, University of Vermont, USA. Transgenic mice were maintained as heterozygous. Balb/c mice, two independent lines of C57BL/6 *Nlrp1b1*-transgenic mice and their Wt littermates were feed with a regular chow diet (2018SX; Harlan Teklad Global) or with a high fat diet (D12492, Research diets, Table I) *ad libitum*. Food consumption and weight were recorded weekly. All the experiments involving mice were approved by the Bioethics committee of the Instituto de Biotecnología, Universidad Nacional Autónoma de México and were according to the National Institute of Health (NIH) guidelines.

### Macrophage activation

2 × 10^5^ bone marrow derived macrophages from Balb/c, wild type C57BL/6 or Nlrp1b1-transgenic mice were stimulated with the anthrax lethal toxin (200 ng/ml lethal factor and 1μg/ml protective antigen) for 2 hours. When stimulated with ATP, glucose or palmitic acid, macrophages were previously stimulated with LPS (100 ng/ml) for 4 hours and then exposed to: ATP (5 mM) for 2 hours; or Glucose (30 mM); Sorbitol (30 mM, used as an osmolarity control); or bovine serum albumin (BSA)-Ac.Pal (400 mM Palmitic Acid, 2% BSA, 1% FBS in DMEM), or BSA alone (2% BSA, 1% FBS in DMEM used as a carrier control), for 24 hours. After, the supernatants were harvested and stored at −70 °C, until use.

### 3T3-L1 preadipocytes culture and differentiation

The 3T3-L1 preadipocytes were donated by Dr. Ramón González García-Conde (UAEMor, México). 3T3-L1 cells were maintained according to Chung *et al*., 2010^35^. Cells were cultured in DMEM supplemented with 10% FBS, 1% antibiotic, 1.5 g/L of sodium bicarbonate and 25 mM pyruvate under an atmosphere of 5% CO_2_ at 37 °C. One day post-confluence, 3T3-L1 cells were differentiated in DMEM supplemented with 10% FBS, 5 μg/ml insulin, 0.5 mM 3-isobutyl-1-methylxanthine and 1 μM dexamethasone. Two days after, medium was replaced with DMEM supplemented with 5 μg/ml insulin for up to 2 days, changing the medium every day. On the 5^th^ day of differentiation the cells were used for experiments. Before treatment with recombinant IL-18 (rIL-18), the differentiated 3T3-L1 cells were changed to culture medium in absence of any differentiating agents. One hour later, cells were left untreated or treated with isoproterenol (10μM) or with rIL-18 (10 ng/ml or 50 ng/ml) for the indicated periods of time. Total protein extracts were prepared as described below and stored at −70 °C until use.

### Antibodies

The anti-phosphorylated AKT (Ser 473; No. 9271), anti-AKT (No. 9272), anti-phosphorylated HSL (Ser 660; No. 4126), anti-HSL (No. 4107) and perilipin (No. 9349) antibodies were obtained from Cell Signaling; the anti-caspase-1 (sc-514), anti-IL-1β (sc-1251), anti-ERK2 (sc-154), the anti-IL-18 (sc-7954) and the anti-actin (sc-1615) antibodies were obtained from Santa Cruz Biotechnology.

### Insulin Tolerance Test (ITT) and Glucose Tolerance Test (GTT)

Animals were starved for six hours before the test. Each mouse was given intraperitoneally, 1 mU insulin Humulin® R (ITT) or 1.8 mg of D-glucose (GTT) per gram of body mass. The blood glucose concentration was determined at 0, 15, 30, 60 and 120 min after injection using an Accu-Chek Active® meter.

### Area under the curve (AUC)

The AUC for both the ITT and GTT was calculated using the Tai’s formula:

$$Area=\frac{1}{2}{\sum }_{\iota =\,1}^{n}{\rm{{\rm X}}}\iota -1(\varUpsilon \iota -1+\varUpsilon \iota )$$

where X_1_ = Glucose (mg/dl), $(\varUpsilon \iota -1+\varUpsilon \iota )$=Time (0,15,30,60,120 min)^36^.

### Tissue preparation

Tissues were fixed in 4% paraformaldehyde in phosphate-buffered saline overnight at 4 °C, then incubated in 30% sucrose in PBS overnight at 4 °C; or embedded in OCT; or snap frozen in liquid nitrogen and stored at −80 °C until use. Blood samples obtained by cardiac puncture were kept at room temperature (RT) approximately two hours to promote clot formation. Then, centrifuged at 1,200 rpm for 10 min; the serum was collected and stored at −20 °C. For experiments where activation of insulin signaling pathway was evaluated, mice were injected intraperitoneally with insulin 1 mU Humulin-R® per gram of body weight and sacrificed 5 or 10 min later.

### Adipose and Liver tissue histochemistry

Adipose tissues were fixed in 10% paraformaldehyde and embedded in paraffin. 5 μM-thick sections were stained with hematoxylin and eosin. Analysis of adipocyte histology was performed with ImageJ software according to the manual procedure (http://rsbweb.nih.gov/ij/). Immune cells infiltration in adipose tissue was quantified by calculating the ratio of infiltration on 20 fields (20×), of 3 slides for each individual mice using 5 mice for each group. Immune cells forming crown like-structure per mm^2^ was estimated by counting the number of cells in 3 slides obtained from 5 mice in 10 fields (63×). The liver tissue was mounted in the Tissue-Tek OCT (Sakura Finetek, Torrance, CA) and frozen for sectioning. Liver slides were stained with hematoxylin and eosin. Light microscopic images were acquired using a Zeiss LSM510/UV Axiovert 200 M confocal microscope with Nikon COOLPIX 5000 color camera.

### Immunofluorescence

Peritoneal adipose tissue was embedded in paraffin. Sections (5 μm) were cut onto charged glass slides (Superfrost Plus Yellow) and rehydrated. The heat-induced antigen retrieval was performed using citrate buffer pH 6.0 (10 μM sodium citrate) at 90 °C for 20 minutes. The sections were permeabilized (10 mg/mL BSA, 5% horse serum, 0.02% sodium azide, 0.5% Triton X-100) for 2 hours and incubated with anti-Ly6G (BD), anti-F4/80 (Abcam) or anti-FoxP3 (Biolegend) primary antibodies at RT for 18 hours. The secondary antibodies anti-rat AF 488 (Molecular Probes), anti-rat AF 594 (Jackson ImmunoResearch) and anti-mouse AF 647 (Jackson ImmunoResearch) respectively, were incubated 2 hours at RT. The nuclei were counterstained with Hoechst (Invitrogen) for 10 minutes. The sections were mounted with Vectashield (Vector Laboratories) and acquired on a Nikon Ti Eclipse inverted confocal microscope (Nikon Corporation) using NIS Elements v.4.50 and analyzed using ImageJ Software (ImageJ software, National Institutes of Health; http://rsbweb.nih.gov/ij/).

### Total protein extracts and immunoblotting

Liver and muscle samples were thawed in 400–600 μL of lysis buffer (20 mM Tris pH 7.4, 137 mM NaCl, 25 mM β-glycerophosphate pH 7.4, 2 mM PPiNa, 2 mM EDTA pH 7.4, 1% Triton X-100, 10% glycerol) supplemented with complete protease inhibitor (Roche) and phosphatase inhibitors (200 mM Na_3_VO_4_, 0.1 mM DTT, 1 mM PMSF). The tissue was then sonicated and the homogenate was incubated on ice for 10 min. Frozen adipose tissue was placed in a mortar with liquid nitrogen and macerated. The macerated was collected in eppendorf tubes and 400–600 μl of lysis buffer were added. After 10 min incubation on ice, samples were centrifuged at 14,500 rpm at 4 °C, the supernatants were recovered and stored at −80 °C. Immunoblot was performed as described^37^. The antibody-antigen interactions were visualized by chemiluminescence using a LI-COR Biosciences instrument. Densitometry was performed using the Image Studio Software Version 5.2.5.

### ELISA

Circulating insulin levels were quantified in blood sera using the Rat/Mouse Insulin ELISA Kit EMD Millipore (Cat. # EZRMI-13K), following the manufacture instructions. IL-1β levels in culture supernatants or in plasma were quantified using the IL-1β ELISA MAX^TM^ Deluxe Sets Biolegend (Cat. # 432605) following the manufactures instructions.

### Lipids determination

Fasting plasma glucose (FPG), total cholesterol (CHOD) and triglycerides (TG) were determined by an enzymatic colorimetric assay using glucose oxidase test and cholesterol oxidase, 4-aminophenazone (Roche-Cobas C111 kit, Roche Diagnostic USA). The intra- and inter-assay variation coefficient was 2.5% and 2.8%, respectively. High-density lipoprotein cholesterol (HDL-C) was measured using enzymatic direct methods: PEG cholesterol esterase and PEG cholesterol-oxidase (HDL- C plus 3er generation, Roche-Cobas C111 kit, Roche Diagnostic USA). Low-density cholesterol (LDL-C) was calculated by the Anandaraja’s formula in individuals with serum TG <150.0 mg/dl^38^.

### Statistical analysis

The results are presented as the mean ± standard error of the mean. The data were subjected to one-way ANOVA followed by Tukey’s multiple comparison test, using graph Prism 6. Significant differences were considered with a p value  <0.05.

## Supplementary information

Supplementary Dataset1

Supplementary Materials and Methods
