# Supplementary material for: Nlrp1b1 negatively modulates obesity-induced inflammation by promoting IL-18 production
Source: Sci Rep. 2019 Sep 25;9:13815. doi: 10.1038/s41598-019-49546-7 (PMC6761090; doi:10.1038/s41598-019-49546-7)
Supplement: Supplementary file 1 — Supplementary Dataset1 [file 41598_2019_49546_MOESM1_ESM.pdf]

Nlrp1b1 negatively modulates obesity-induced inflammation by promoting IL-18 production

Salazar-León Jonathan, Valdez-Hernández Ana Laura, García-Jiménez Sara, Román-Domínguez Luis, Huanosta-Murillo Enrique, Bonifaz Laura C., Pérez-Martínez Leonor and Pedraza-Alva Gustavo

SUPPLEMENTARY FIGURES

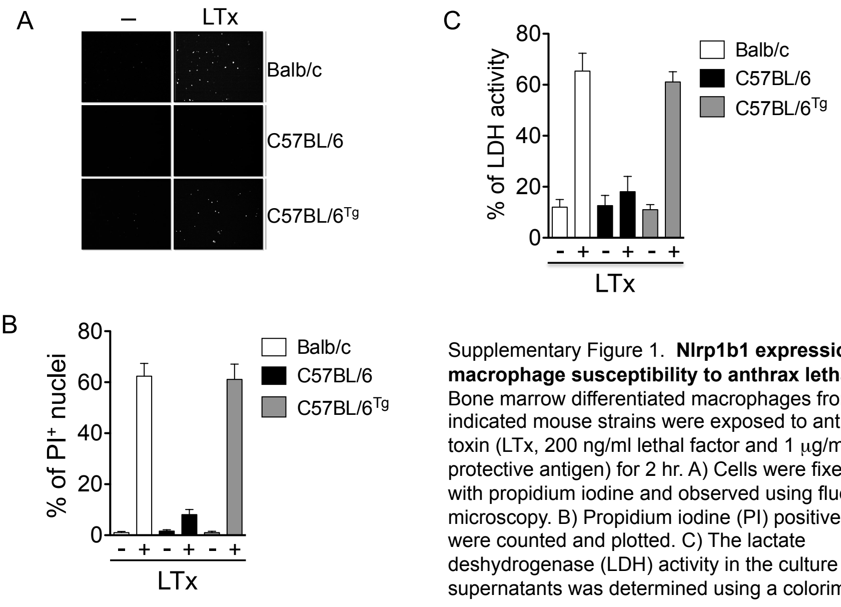

Supplementary Figure 1. **Nlrp1b1 expression confers macrophage susceptibility to anthrax lethal toxin**. Bone marrow differentiated macrophages from the indicated mouse strains were exposed to anthrax lethal toxin (LTx, 200 ng/ml lethal factor and 1 μg/ml protective antigen) for 2 hr. A) Cells were fixed, stained with propidium iodine and observed using fluorescence microscopy. B) Propidium iodine (PI) positive nuclei were counted and plotted. C) The lactate deshydrogenase (LDH) activity in the culture supernatants was determined using a colorimetric assay.

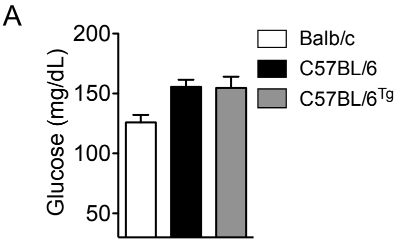

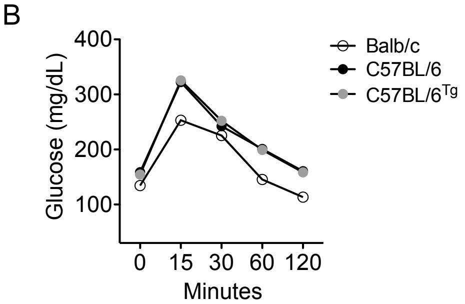

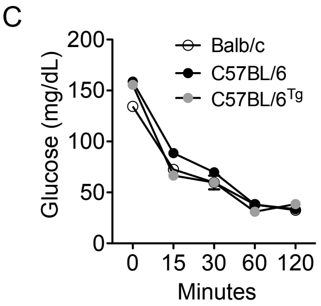

Supplementary Figure 2. **Nlrp1b1 expression does not alter C57BL/6 mice glucose metabolism**. A) Basal blood glucose levels. Mice from the different strains were starved for six hours and basal blood glucose levels were determined as described in materials and methods. B) Intraperitoneal GTT. After six hours starvation mice from the different strains received 1.8 mg of D-glucose per gram of body mass via intraperitoneal and glucose levels were determined at the indicated time points after glucose administration. C) Intraperitoneal ITT. After six hours starvation, mice from the different strains received insulin (1 mU/gr) via intraperitoneal and glucose levels were determined at the indicated time points after insulin administration.

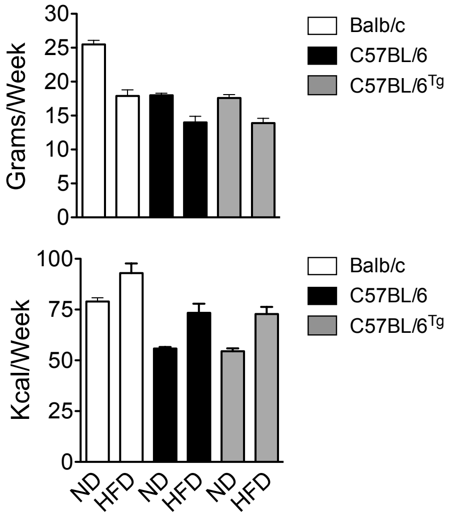

Supplementary Figure 3. **The Nlrp1b1 gene does not affect food intake**. Mice were fed with regular chow (ND) or with a HFD for three months. Food consumption (upper panel) and calories intake (lower panel) were calculated. Data represent the food or Kcal consumed per mouse in a week.

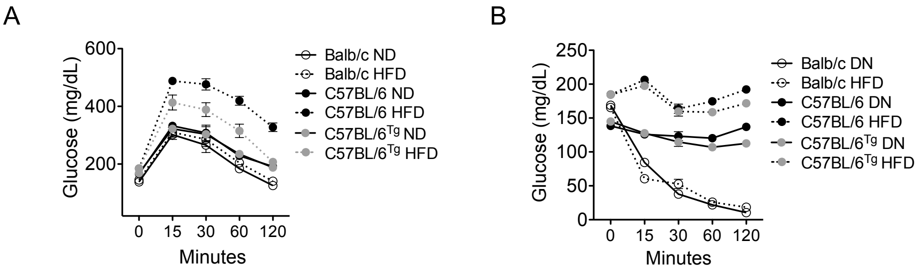

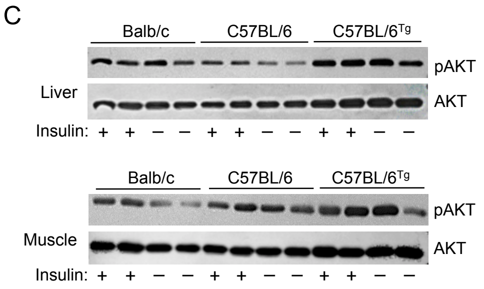

Supplementary Figure 4. **The Nlrp1b1 gene attenuates obesity-induced glucose tolerance and improves insulin sensitivity in obese mice** Mice were fed with regular chow (ND) or with high fat diet (HFD) for three months. Mice from the different strains were starved for six hours. Starved mice received 1.8 mg of D-glucose for GTT (A) or insulin (1 mU) for ITT (B), per body gram via intraperitoneal. Glucose levels were determined at the indicated time points after injection. Phosphorylated AKT (pAKT) levels were evaluated by immunoblot using specific antibodies, in liver (C, upper panel) or muscle (C, lower panel) total protein extracts prepared from mice fed with the HFD, 10 min after the administration of 1 mU of insulin per body gram via intraperitoneal. Total AKT levels were used as loading control.

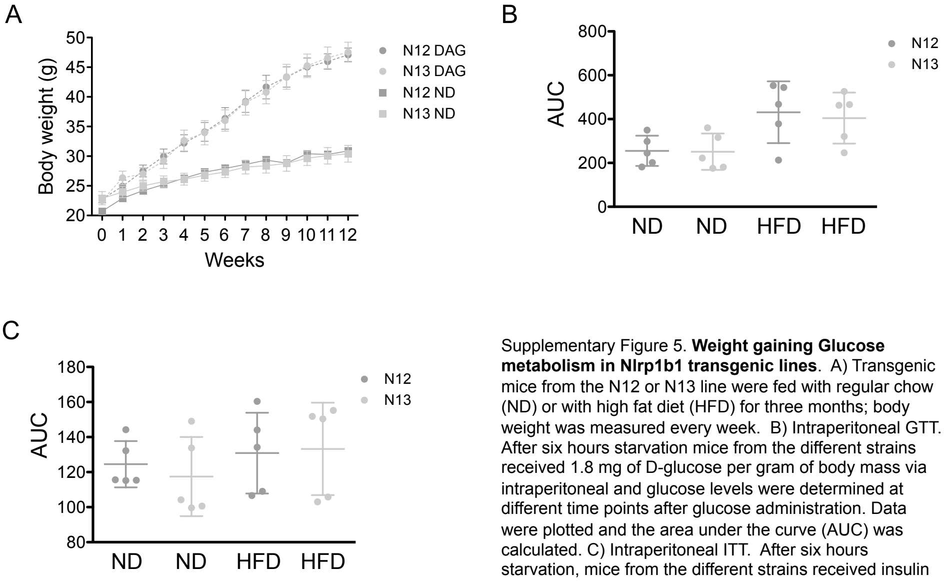

Supplementary Figure 5. **Weight gaining Glucose metabolism in Nlrp1b1 transgenic lines**. A) Transgenic mice from the N12 or N13 line were fed with regular chow (ND) or with high fat diet (HFD) for three months; body weight was measured every week. B) Intraperitoneal GTT. After six hours starvation mice from the different strains received 1.8 mg of D-glucose per gram of body mass via intraperitoneal and glucose levels were determined at different time points after glucose administration. Data were plotted and the area under the curve (AUC) was calculated. C) Intraperitoneal ITT. After six hours starvation, mice from the different strains received insulin (1 mU/gr) via intraperitoneal and glucose levels were determined at different time points after insulin administration. Data were plotted and the area under the curve (AUC) was calculated.

A

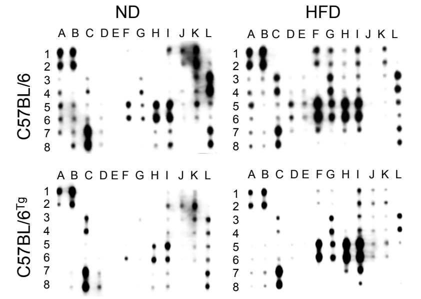

C

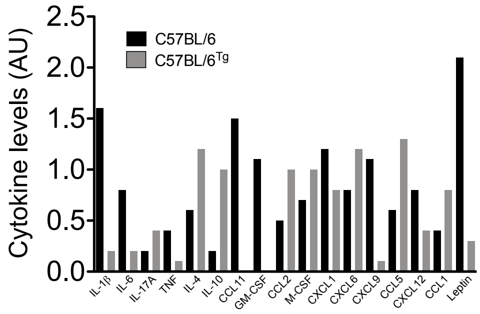

B

Each antibody is spotted in duplicate vertically	A	B	C	D	E	F	G	H	I	J	K	L
1 2	POS	POS	NEG	NEG	BLANK	BLC (CXCL13)	CD30 Ligand (TNFSF8)	Eotaxin-1 (CCL11)	Eotaxin-2 (CCL24)	Fas Ligand (TNFSF6)	Fractalkine (TNFSF8)	GCSF (CCL11)
3 4	GM-CSF	IFN-gamma	IL-1 alpha (IL-1 F1)	IL-1 beta (IL-1 F2)	IL-2	IL-3	IL-4	IL-6	IL-9	IL-10	IL-12 p40/p70	IL-12 p70
5 6	IL-13	IL-17A	I-TAC (CXCL1)	KC (CXCL1)	Leptin	LIX	XCL1	MCP-1 (CCL2)	M-CSF	MIG (CXCL9)	MIP-1 alpha (CCL3)	MIP-1 gamma
7 8	RANTES (CCL5)	SDF-1 alpha (CXCL12 a)	I-309 (TCA-3/CCL1)	TECK (CCL25)	TIMP-1	TIMP-2	TNF alpha	TNF RI (TNFRSF1A)	TNF RII (TNSFRSF1A)	BLANK	BLANK	POS

Supplementary Figure 6. **The *Nlrp1b1* gene attenuates the inflammatory process in the adipose tissue resulting from a HFD consumption**. A) Protein extracts were prepared from the adipose tissue from wild type C57BL/6 or *Nlrp1b1*-transgenic (C57BL/6^{Tg}) mice fed with a normal diet (ND) or a high fat diet (HFD) and the cytokine levels were evaluated using the Mouse inflammation antibody array C1 RayBio® C-Series as indicated under materials and methods. B) The location for each cytokine on the membrane is shown. C) Cytokine levels in the adipose tissues from obese wild type C57BL/6 or Nlrp1b1-transgenice (C57BL/6^{Tg}). Densitometry values represent the mean of two independent membranes probed with extracts from two obese mice of each genotype.

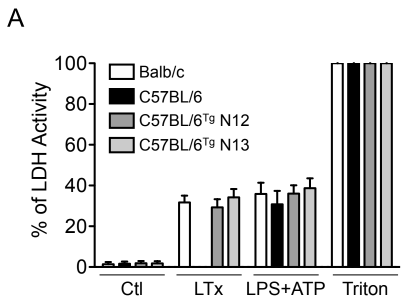

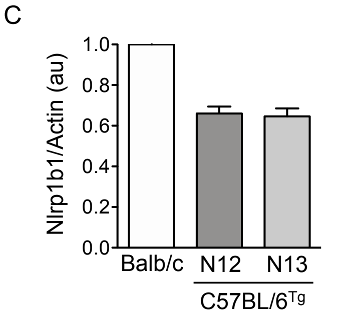

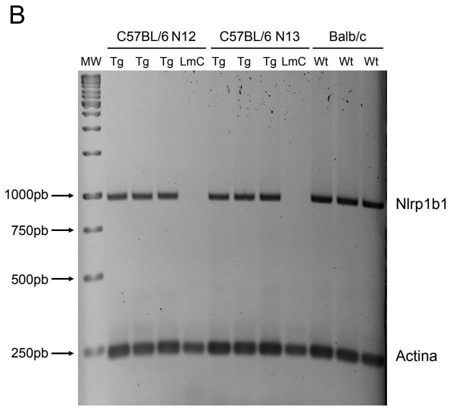

Supplementary Figure 7. **The Nlrp1b1 gene dosage does not impair NLRP3 inflammasome activation**. A) Bone morrow derived macrophages from Balb/c, C57BL/6 litter control and from two independent C57BL/6 *Nlrp1b1* transgenic lines (N12 and N13) were left untreated (Ctl) or exposed to exposed to anthrax lethal toxin (LTx, 200 ng/ml lethal factor and 1 μg/ml protective antigen) for 2 hr, or to LPS (100 ng/ml) for 4hr and then treated with ATP (5mM) for 2 hr. The lactate deshydrogenase (LDH) activity in the culture supernatant was determined using a colorimetric assay. B) Genomic DNA was prepared from both *Nlrp1b1* transgenic lines as well as from their corresponding negative litter mate controls (LmC) or from Balb/c mice. The Nlrp1b1 gene dosage in the different Nlrp1b1 transgenic lines was determined by PCR. DNA products were resolved in agarose gels. C) The *Nlrp1b1* and *Actin* levels were calculated from the densitometry values of their corresponding PCR DNA products shown in panel B. *Nlrp1b1/Actin* ratio found in Balb/c mice (diploid genome) was adjusted to 1.

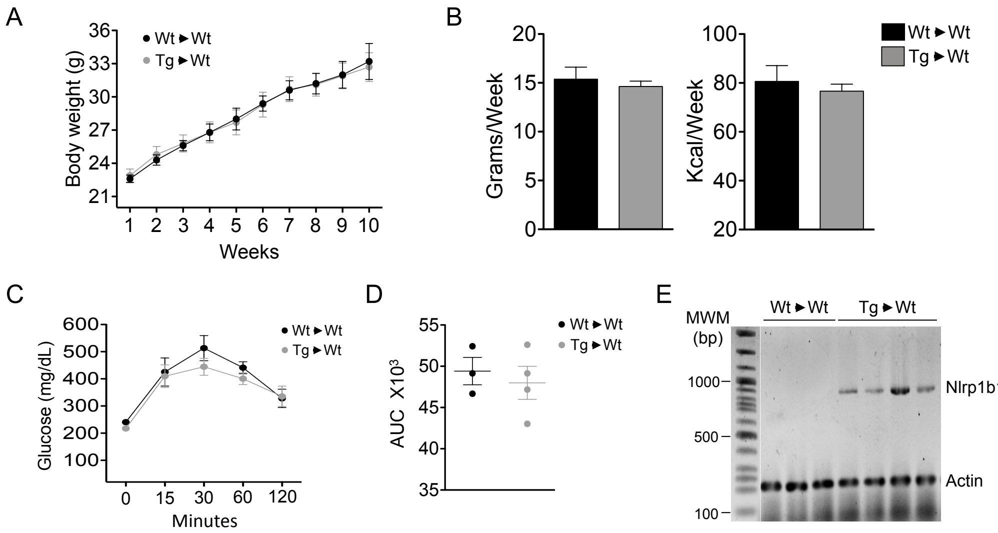

Supplementary Figure 8. **The effects of the *Nlrp1b1* gene on glucose metabolism does not result from its expression in the hematopoietic compartment.** Lethally radiated wild type (Wt) C57BL/6 mice received Wt (Wt➔Wt) or *Nlrp1b1*-transgenic (Tg➔Wt) bone marrow cells. A) Mice were then fed with HFD for teen weeks and weighted every week. B) Food consumption (left panel) and calories intake (right panel) were calculated. Data represent the food or Kcal consumed per mouse in a week. C.) Intraperitoneal GTT. After six hours starvation mice from the different strains received 1.8 mg of D-glucose per gram of body mass via intraperitoneal and glucose levels were determined at the indicated time points after glucose administration. D) GTT area under the curve (AUC). E) The bone marrow engraftment was confirmed by evaluating the presence of transgenic bone marrow cells in the Wt mice. At the end of the experiment mice were sacrifice and bone marrow DNA was prepared, the presence *Nlrp1b1* transgene was determined by PCR, as described under material and method. Actin was used as internal control. The standard deviation of the mean is shown in the graphs. Wt (C57BL/6) n=3 and *Nlrp1b1*-Tg (Tg) n=4.
