# Supplementary material for: Nlrp1b1 negatively modulates obesity-induced inflammation by promoting IL-18 production
Source: Sci Rep. 2019 Sep 25;9:13815. doi: 10.1038/s41598-019-49546-7 (PMC6761090; doi:10.1038/s41598-019-49546-7)
Supplement: Supplementary file 2 — Supplementary Materials and Methods [file 41598_2019_49546_MOESM2_ESM.pdf]

Nlrp1b1 negatively modulates obesity-induced inflammation by promoting IL-18 production

Salazar-León Jonathan, Valdez-Hernández Ana Laura, García-Jiménez Sara, Román-Domínguez Luis, Huanosta-Murillo Enrique, Bonifaz Laura C., Pérez-Martínez Leonor and Pedraza-Alva Gustavo

SUPPLEMENTARY MATERIALS AND METHODS

Genomic DNA Extraction and PCR

Genomic DNA was extracted from mouse-tails or bone marrow to detect the presence of the *Nlrp1b1* gene in the transgenic C57BL/6 mice or in the bone marrow transplants of the recipient wild type C57BL/6 mice, respectively. Tissue was digested in lysis buffer (50 mM Tris pH 8.0, 100 mM EDTA pH 8.0, 100 mM NaCl, 4% SDS, 5 mg/ml pronase) overnight at 56ºC. Samples were chilled on ice, RNAse (25 μg) was added and incubated at 37ºC for 30 min. DNA was ethanol precipitated and resuspended in H_2O, quantified and stored at -20ºC until use.
PCR reactions were performed using 20 ng of genomic DNA and 20 pM of specific oligonucleotides. *Nlrp1b1:* forward (TTG ACA GAG AGT GAG GAA CCT ATA CC) and reverse (GGG ACC ATC ATT TGT GGC ATC) for 35 cycles (1min at 94ºC, 1min at 68ºC, 1.5 min at 72ºC) and a final extension at 72ºC for 10 min. *Actin*: forward (GGG TCA GAA GGA CTC CTA TG) and reverse (GGT CTC AAA CAT GAT CTG GG) for 30 cycles (45 s at 95ºC, 30 s at 60ºC, 1 min at 72ºC) and a final extension at 72ºC for 7 min. DNA fragments were resolved by agarose gel electrophoresis and visualized by ethidium bromide staining.

Anthrax Lethal toxin and LPS/ATP treatment

Bone marrow cells were extracted from the tibiae and femurs of the different mouse strains. Bone marrow cells ($5X10^6$) were cultured onto non-treated culture plates in DMEM containing 10% FCS, 2 mM L-glutamine, 1U/mL penicillin and 1U/mL streptomycin, supplemented with 30% of the supernatant from G-MCSF producing L-929 cells, for seven days. Macrophages were recovered and plated on 24 wells plates ($2X10^5$/well) and cultured in RPMI medium containing 2% FCS, 2 mM L-glutamine, 1U/mL penicillin and 1U/mL streptomycin for 3 days. For the challenge with the anthrax toxin (TxLt), macrophages were incubated for 2 hours in the absence or in the presence of 20, 200 or 400 mg/ml of the lethal factor and 1 mg/ml of protective antigen. To evaluate inflammasome activation in response to ATP, macrophages were treated with LPS (100 ng/ml) for 4 hours and then exposed to ATP (5mM) for two hours. Viability was determined by measuring Lactate Dehydrogenase (LDH) activity in the culture supernatants and by propidium iodide staining. LDH activity was determined using the CytoTox 96® Non-Radioactive Cytotoxicity Assay Kit (Promega) following the manufacturer's protocol. For propidium iodine staining, macrophages were washed with PBS and incubated with propidium iodide (5 mg/ml) for 5 min, macrophages were then washed, fixed in 4% paraformaldehyde and observed under a Olympus inverted microscope ix8i epifluorescence Cooled PE2.

Bone marrow cells transplant

8-week-old wild type C57BL/6 mice were irradiated with a lethal dose using a GammaCell-1000E Cesium-137 irradiator. Subsequently, mice were inoculated with

$5X10^5$ bone marrow cells from either wild type C57BL/6 or *Nlrp1b1*-transgenic mice. Five weeks later, animals were fed with a high-fat diet for ten weeks. After this period of time, glucose tolerance test was performed as described under materials and methods. Animals were sacrificed, and genomic DNA was obtained from bone marrow cells. The presence of the *Nlrp1b1* transgene was determined by PCR.

Adipose tissue inflammatory profile

Protein extracts from the adipose tissue were prepared as described under materials and methods. The adipose tissue inflammatory state from mice fed with a normal or high fat diet was determined by the mouse inflammation antibody array C1 RayBio® C-Series (#AAM-INF-1-4). Arrays were probed with 250 μg of protein extracts following the manufacturer's instructions. The antibody-antigen interactions were visualized by chemiluminescence using a LI-COR Biosciences instrument. Densitometry was performed using the Image Studio Software Version 5.2.5.
